# *Dillenia Suffruticosa* Extract Inhibits Proliferation of Human Breast Cancer Cell Lines (MCF-7 and MDA-MB-231) via Induction of G_2_/M Arrest and Apoptosis

**DOI:** 10.3390/molecules181113320

**Published:** 2013-10-29

**Authors:** Nurdin Armania, Latifah Saiful Yazan, Intan Safinar Ismail, Jhi Biau Foo, Yim Sim Tor, Nurshafini Ishak, Norsharina Ismail, Maznah Ismail

**Affiliations:** 1 Laboratory of Molecular Biomedicine, Institute of Bioscience, Universiti Putra Malaysia, 43400 UPM Serdang, Selangor, Malaysia; 2 Department of Biomedical Science, Faculty of Medicine and Health Sciences, Universiti Putra Malaysia, 43400 UPM Serdang, Selangor, Malaysia; 3 Laboratory of Natural Product, Institute of Bioscience, Universiti Putra Malaysia, 43400 UPM Serdang, Selangor, Malaysia; 4 Department of Chemistry, Faculty of Science, Universiti Putra Malaysia, 43400 UPM Serdang, Selangor, Malaysia; 5 School of Medical Science, Health Campus, Universiti Sains Malaysia, 16150 Kubang Kerian, Kelantan, Malaysia; 6 Department of Nutrition and Dietetics, Faculty of Medicine and Health Sciences, Universiti Putra Malaysia, 43400 UPM Serdang, Selangor, Malaysia

**Keywords:** *Dillenia suffruticosa*, apoptosis, cell cycle arrest, fractionation

## Abstract

The present research was designed to evaluate the anticancer properties of *Dillenia suffruticosa* extract. Our focus was on the mode of cell death and cell cycle arrest induced in breast cancer cells by the active fractions (designated as D/F4, D/F5 and EA/P2) derived from chromatographic fractionation of *D. suffruticosa* extracts. The results showed that the active fractions are more cytotoxic towards MCF-7 (estrogen positive breast cancer cells) and MDA-MB-231 (estrogen negative breast cancer cells) as compared to other selected cancer cell lines that included HeLa, A459 and CaOV3. The induction of cell death through apoptosis by the active fractions on the breast cancer cells was confirmed by Annexin V-FITC and PI staining. Cell cycle analysis revealed that D/F4 and EA/P2 induced G2/M phase cell cycle arrest in MCF-7 cells. On the other hand, MDA-MB-231 cells treated with D/F4 and D/F5 accumulated in the sub-G_1_ phase without cell cycle arrest, suggesting the induction of cell death through apoptosis. The data suggest that the active fractions of *D. suffruticosa* extract eliminated breast cancer cells through induction of apoptosis and cell cycle arrest. The reason why MCF-7 was more sensitive towards the treatment than MDA-MB-231 remains unclear. This warrants further work, especially on the role of hormones in response towards cytotoxic agents. In addition, more studies on the mechanisms underlying the induction of apoptosis and cell cycle arrest by the plant extract also need to be carried out.

## 1. Introduction

Characterized by abnormal growth and development of normal cells beyond their natural boundaries, cancer has been claimed to become one of the most complex dynamic human diseases [1]. As the most prevalent and life threatening diseases after heart diseases, cancer causes worldwide approximately 12.7 million and 7.6 million incidences and deaths, respectively [2].

There was no single cause for any particular type of cancer, and therefore cancer is regarded as multifactorial diseases involving both genetics and environmental factors. The genome integrity is tightly regulated by cell cycle to stop any genetic alteration from being passed through to subsequent generations [3]. However, deregulations of cell cycle checkpoints including those of the G_1_/S and G_2_/M phases have been documented to be associated with malignancy [4]. Cell cycle arrest provides an opportunity for DNA repair to occur, hence inhibiting replication of the damaged template [5]. It is regarded as one of the effective strategies for eliminating the cancer cells [6].

Besides cell cycle, apoptosis or programmed cell death is also another key strategy for eliminating cancerous cells [7]. It acts as a protective mechanism that destroys potentially harmful or damaged cells before manifestation of malignancy [8], without activating the inflammatory responses [9]. Many chemotherapeutic drugs including cisplatin [10,11], tamoxifen [12] and doxorubicin [13] cause cell cycle arrest and induce apoptosis in eliminating the neoplastic cells.

Despite the effectiveness of current cancer chemotherapeutic drugs, the presence of adverse side effects have urged demand for development of new therapeutic agents. In this task, natural product-derived drugs have been preferred since they are claimed to be an important source for drug lead candidates. *Dillenia suffruticosa* or “*Simpoh air*” (local name in Malaysia) is a medium-size tree (up to 6 meters in height) naturally found in evergreen forests ranging from West Malaysia to the Philippines, Indonesia and Brunei Darussalam. It displays large oval leaves, flowers with five thin yellow petals and pink star-shaped fruit capsules [14]. It has been ethno-medically reported to relieve rheumatism [15], promote wound healing and treat fever [16].

In addition, the fruit of the plant has been used traditionally as a treatment for cancerous growths [17]. Armania *et al*. [18] reported in 2013 that the root part of *D. suffruticosa* was the most cytotoxic against a few selected human cancer cell lines as compared to other parts of the plant including the fruit. Further analysis of dichloromethane (DCM) and ethyl acetate (EtOAc) extracts of the root of the plant showed the highest cytotoxicity towards selected cancer cell lines, including breast cancer cell lines (MDA-MB-231 and MCF-7). Hence, this study evaluated the cytotoxic activity of the active fractions derived from chromatographic fractionation of *D. suffruticosa* extracts (DCM and EtOAc) on different cancer cell lines, and mode of cell death and cell cycle arrest induced in breast cancer cell lines (MCF-7 and MDA-MB-231) by the fractions.

## 2. Results and Discussion

### 2.1. Cytotoxicity of DCM and EtOAc Fractions of *D. Suffruticosa* towards Selected Cancer Cell Lines

Previously, DCM and EtOAc extracts of *D. suffruticosa* were found to be the most cytotoxic towards the selected cancer cell lines as compared to other extracts [18]. Consequently, fractionation of these DCM and EtOAc extracts was carried out in this study, and yielded a total of 17 and 2 fractions, respectively, as illustrated in Table 1 and Table 2, respectively. For our cytotoxic screening, in addition to human cervical adenocarcinoma (HeLa, IC_50_ = 19.67 ± 0.58 µg/mL) and human ovarian adenocarcinoma (CaOV3, IC_50_ = 12.33 ± 0.58 µg/mL) [18], human breast adenocarcinoma (MCF-7 and MDA-MB-231) and human lung carcinoma (A549) cell lines were also included since they are among the most common cancers worldwide [2].

**Table 1:** Yield of DCM fractions of *D. suffruticosa* extract.

Fraction	TLC Mobile Phase	TLC R*f* Values	Weight (mg)	Yield (%)
D/F1	Hexane-EA, 9:1 v/v	0.90, 0.98	17.90	0.90
D/F2	Hexane-EA, 9:1 v/v	0.85, 0.90, 0.94, 0.98	89.40	4.47
D/F3	Hexane-EA, 9:1 v/v	0.15, 0.26, 0.48, 0.52, 0.66, 0.74, 0.80, 0.89	139.30	6.97
**D/F4**	**Hexane-EA**, **4:1** ** v/v**	**0.54**, **0.68**, **0.77**, **0.92**, **0.95** *	**606.70**	**30.34**
**D/F5**	**Hexane-EA**, **3:2 v/v**	**0.25**, **0.48**, **0.60**, **0.72** *	**571.00**	**28.55**
D/F6	Hexane-EA, 1:1 v/v	0.43, 0.49, 0.59, 0.67, 0.78, 0.85	141.80	7.09
D/F7	Hexane-EA, 1:1 v/v	0.23, 0.39, 0.48, 0.61, 0.94, 0.97	41.20	2.06
D/F8	Hexane-EA, 2:3 v/v	0.31, 0.62, 0.74, 0.92	9.30	0.47
D/F9	Hexane-EA, 2:3 v/v	0.24, 0.35, 0.95	30.20	1.51
D/F10	EA, 100%	0.48, 0.93	8.80	0.44
D/F11	EA, 100%	0.34, 0.41, 0.62, 0.77, 0.91	12.10	0.61
D/F12	EA-MeOH, 98:2 v/v	0.06, 0.69, 0.89	32.20	1.61
D/F13	EA-MeOH, 97:3 v/v	0.70, 0.80, 0.90	41.40	2.07
D/F14	EA-MeOH, 95:5 v/v	0.74, 0.79, 0.93	13.00	0.65
D/F15	EA-MeOH, 9:1 v/v	0.68, 0.77, 0.80, 0.90	18.50	0.93
D/F16	EA-MeOH, 9:1 v/v	0.12, 0.69, 0.77, 0.83, 0.89	64.30	3.22
D/F17	EA-MeOH, 9:1 v/v	0.11, 0.82, 0.89	10.20	0.51

* with tailing effect.

Two out of the 17 fractions designated as D/F4 and D/F5 were found to be the most cytotoxic towards the selected cancer cell lines, with IC_50_ values ranging from 9.50 ± 0.50 to 23.00 ± 2.65 µg/mL and 17.67 ± 0.58 to 22.00 ± 0.58 µg/mL, respectively (Table 3). As the main objective of this study was to evaluate the cytotoxicity of the fractions towards cancer cell lines, only those fractions (D/F4 and D/F5) with IC_50_ values below 30 µg/mL were subjected to cytotoxic activity determination towards the non-tumorigenic Swiss mouse embryo fibroblast cells (3T3 F442A). The yield of some other fractions was too limited as compared to the active fractions for the experiment purposes (Table 1). 

**Table 2:** Cytotoxicity of EtOAc fractions of *D. suffruticosa* on selected cancer cell lines.

Cell Line/Fraction	IC_50_ (µg/mL)	
	EA/P1	EA/P2
MDA-MB-231	>150	70.00 ± 1.00
MCF-7	>150	34.33 ± 1.53
CaOV3	>150	53.67 ± 0.58
HeLa	>150	140.67 ± 2.08
A549	>150	>150
3T3	>150	94.67 ± 2.31

Cytotoxicity after 72 h of incubation as determined by MTT assay represented as IC_50_ value. Non-tumorigenic cell line (3T3 F442A) was also included. The IC_50_ is the average value of three independent experiments ± SD.

**Table 3:** Cytotoxicity of DCM fractions of *D. suffruticosa* on selected cancer and non-tumorigenic cell lines.

Fraction/Cell line	IC_50_ (µg/mL)					
	MDA-MB-231	MCF-7	HeLa	CaOV3	A549	3T3
D/F1	>150	>150	>150	>150	>150	NA
D/F2	>150	>150	>150	>150	>150	NA
D/F3	37.33 ± 6.43	58.33 ± 0.58	50.67 ± 0.58	63.33 ± 0.58	91.00 ± 1.73	NA
**D/F4**	**11.67** ± **0.58**	**9.50** ± **0.50**	**21.00** ± **0.57**	**20.33** ± **1.53**	**23.00** ± **2.65**	18.00 ± 1.15
**D/F5**	**17.67** ± **0.58**	**18.33** ± **0.58**	**19.00** ± **1.00**	**22.00** ± **0.58**	**21.00** ± **0.58**	18.00 ± 0.57
D/F6	56.00 ± 2.00	69.00 ± 1.73	59.00 ± 1.00	43.00 ± 1.00	41.33 ± 1.15	NA
D/F7	75.67 ± 1.53	122.67 ± 3.06	93.33 ± 2.08	91.22 ± 1.15	90 ± 2	NA
D/F8	>150	>150	118.33 ± 1.53	>150	>150	NA
D/F9	>150	>150	>150	>150	>150	NA
D/F10	>150	>150	>150	>150	>150	NA
D/F11	>150	42.33 ± 3.21	133.33 ± 1.53	141.67 ± 1.53	>150	NA
D/F12	>150	88.67 ± 1.15	117.33 ± 1.53	125.67 ± 3.06	114.76 ± 2.31	NA
D/F13	105.00 ± 2.65	111.00 ± 1.00	93.67 ± 0.58	95.67 ± 0.58	88.33 ± 2.52	NA
D/F14	146.67 ± 1.53	147.33 ± 2.08	>150	>150	146.00 ± 0.58	NA
D/F15	>150	>150	>150	>150	>150	NA
D/F16	>150	>150	>150	>150	>150	NA
D/F17	114.67 ± 3.51	>150	>150	>150	>150	NA

Cytotoxicity after 72 h of incubation as determined by MTT assay represented as IC_50_ value. The IC_50_ is the average value of three independent experiments ± SD. NA: Not applicable.

Even though it is crucial for any plant extract to exhibit significant cytotoxicity in cancer cell lines to be considered as good anticancer agent, such activity is ideally only for the cancer cells and not the normal ones. Nevertheless, it was found that D/F4 and D/F5 were also cytotoxic towards 3T3 F442A with IC_50_ of 18.00 ± 1.15 and 18.00 ± 0.57 µg/mL, respectively (Table 3). As shown, there was a significant difference (*p* < 0.05) in the IC_50_ values of different cancer cell lines after treatment with the active fractions (D/F4 and D/F5) as compared to the non-tumorigenic cell lines (3T3 F442A). This indicates lack of selectivity in the cytotoxicity between cancer and non-tumorigenic cell by the extracts.

As shown in Table 2, EA/P2 has better cytotoxic activity towards the selected cancer cell lines as compared to EA/P1. This indicates that the polar compounds which are mainly in fraction EA/P2 of the EtOAc extract are major contributors to the cytotoxic properties of the extract [18]. The solvent-solvent extraction using hexane will extract out the non-polar compounds. The EA/P2 fraction was found to be most cytotoxic towards MCF-7 cell line (IC_50_ = 34.33 ± 1.53 µg/mL). Besides that, EA/P2 was found to be less cytotoxic towards the non-tumorigenic compared to cancer cells.

As also shown in Table 2, the growth inhibitory activities of EA/P2 towards MCF-7 were 3-fold higher compared to 3T3 F442A. Based on that, the active fractions of DCM (D/F4 and D/F5) and EtOAc extract (EA/P2) were further analyzed for the mode of cell death and their effects on the cell cycle. However, given the broad cytotoxicity across the different cancer types, only human breast adenocarcinoma cell lines (MCF-7 and MDA-MB-231) were selected for that purpose.

### 2.2. Morphological Changes of MCF-7 and MDA-MB-231 Cells Treated with Active Fractions of *D. Suffruticosa*

**Figure 1:**
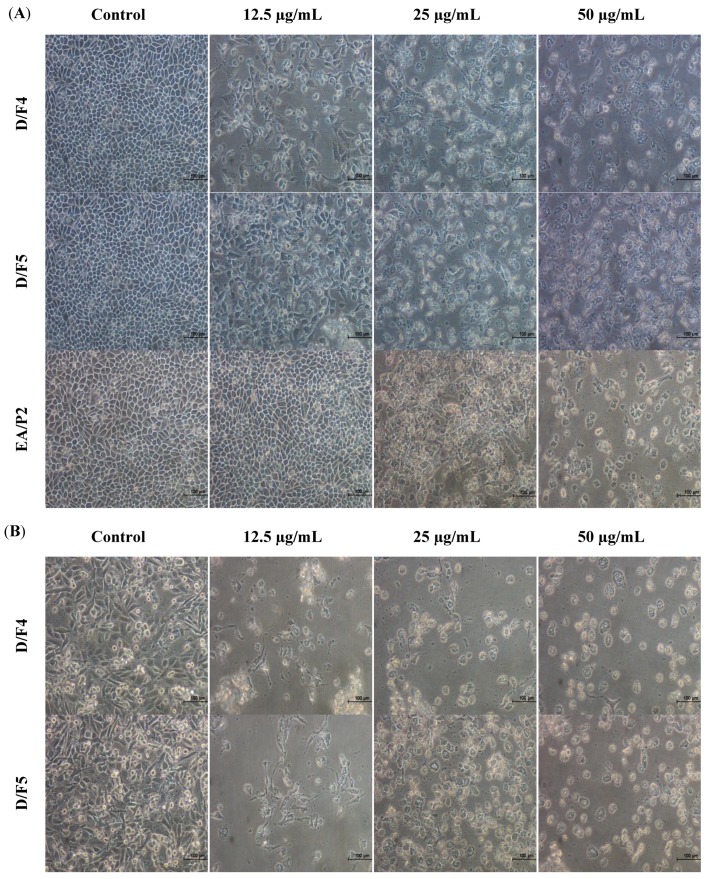
Morphological changes of (**A**) MCF-7 and (**B**) MDA-MB-231 cells treated with different concentrations of active fraction of D/F4, D/F5 and EA/P2 of *D. suffruticosa* for 72 h viewed under an inverted light microscope (200× magnification). Reduce in cell population was noted with the increase in the concentration of the treatment as compared to the control (untreated cells).

**Figure 2:**
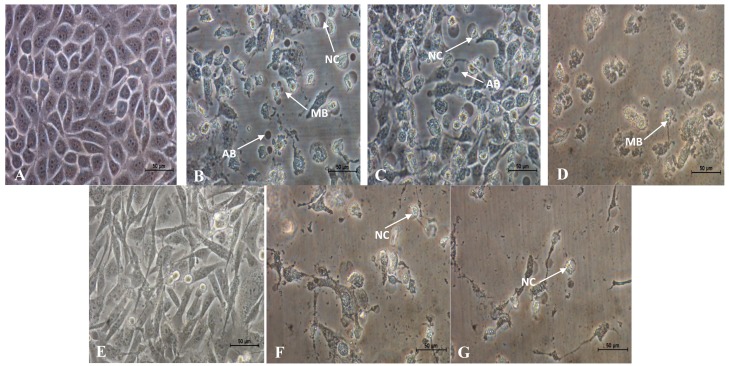
Close-up views of MCF-7(**B**, **C** and **D**) and MDA-MB-231(**F** and **G**) cells treated with (**B** and **F**) 12.5 µg/mL of D/F4, (**C** and **G**) 12.5 µg/mL of D/F5 and (**D**) 50.0 µg/mL of EA/P2 of *D. suffruticosa* for 72 h viewed under an inverted light microscope. The cells showed characteristics of apoptosis such as nuclear compaction (NC), apoptotic bodies (AB) and membrane blebbing (MB). A and E shows the untreated MCF-7 and MDA-MB-231, respectively (400× magnification).

As cell death can be divided into necrosis and apoptosis, it is essential to determine the mode of cell death induced by the active fractions of *D. suffruticosa*. In this study, the morphological changes of human breast adenocarcinoma cell lines (MCF-7 and MDA-MB-231) untreated and treated with the active fractions (D/F4, D/F5 and EA/P2) of *D. suffruticosa* were observed under an inverted light microscope. The most prominent changes characteristic of apoptosis were observed in the treated cells that include the detachment of the cells from substratum, cell shrinkage, nuclear condensation, membrane blebbing as well as formation of apoptotic bodies [19] as shown in Figure 1 and Figure 2. In contrast, the untreated control cells were evenly distributed on the substratum. Reduction in the cell population was noted with the increase in the concentration of D/F4, D/F5 and EA/P2 (Figure 1).

Apoptosis is mainly initiated by detachment and shrinkage of the cells, separation of blebs (protrusions of the plasma membranes) and finally the formation of apoptotic bodies that are packed densely with cellular organelles and nuclear fragments. *In vivo*, the apoptotic bodies will subsequently be engulfed by macrophages or adjacent cells and thus not go through lysis. In contrast, *in vitro,* apoptotic bodies that are formed will ultimately swell and lyses due to the absence of phagocytes and undergo secondary necrosis [20].

### 2.3. Treatment with D/F4 and D/F5 of *D. Suffruticosa* Induced Apoptosis in Cancer Cell Lines

In this study, the cells treated with the highest concentration (50 µg/mL) of the active fractions of *D. suffruticosa* were observed to undergo necrosis. Nevertheless, the induction of necrosis needs to be confirmed by Annexin V-FITC and PI assay. It is probably not the real necrosis but secondary necrosis. This indicates that even at the highest concentration, the mode of cell death is still the preferable one, apoptosis. During early apoptosis, the externalization of the phospholipid phosphatidylserine (PS) occurs at the cell membrane and could be detected by Annexin A5, an endogenous human protein that has higher affinity to bind in a calcium dependent manner to phosphatidylserine. Therefore, Annexin A5 could be used as probe for detection of apoptosis [21]. 

**Figure 3:**
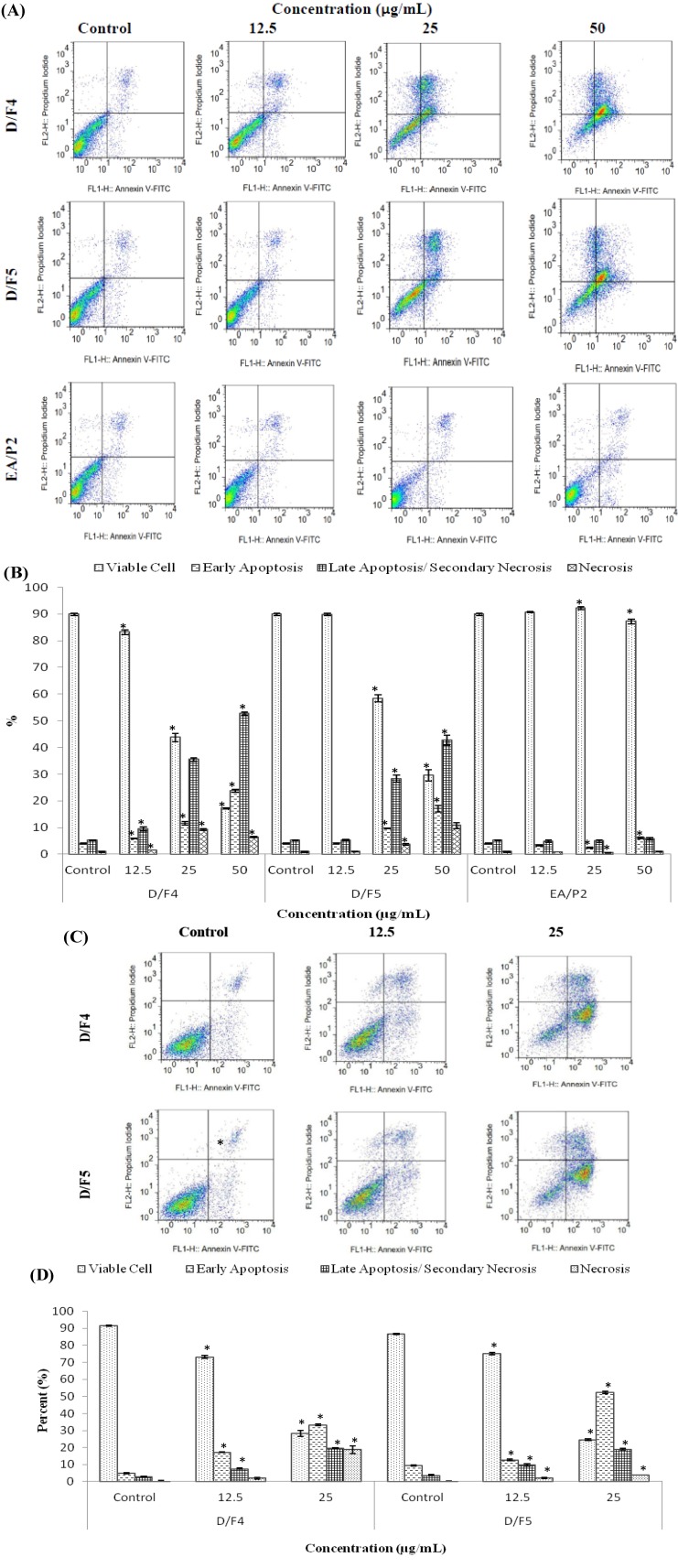
The dot plots (**A**) and the percentage (%) of cell distribution (**B**) of MCF-7 and the dot plots (**C**) and the percentage (%) of cell distribution (**D**) of MDA-MB-231 after treated with the active fractions of *D. suffruticosa* (D/F4, D/F5 and EA/P2) as determined by Annexin V-PI staining. The cells were treated with different concentrations of the active fractions for 24 hours. * *p* < 0.05 as compared to the untreated control cells. The data is the average value of three independent experiments ± SD.

The measurement of apoptosis in the assay is equivalent to the strong affinity of the cells (PS) to bind with Annexin A5 molecules which are conjugated with FITC and analyzed by a flow cytometer [22]. As clearly seen (Figure 3A,B), there was a significant increase (*p* < 0.05) in the population of MCF-7 cells in the early apoptosis, late apoptosis or secondary and necrosis phases after treated with different concentrations of D/F4 and D/F5 as compared to the untreated (control) cells. 

The same trend was also observed in the MDA-MB-231 cells treated with different concentrations of D/F4 and D/F5 (Figure 3C,D). However, MCF-7 cells treated with different concentrations of EA/P2 did not show a similar pattern. It is worthy to highlight that even at the highest concentration of the active fractions of *D. suffruticosa*, the mode of cell death is still apoptosis.

The cell death through apoptosis is more favorable than necrosis due to the induction of the inflammatory response in the affected tissues of necrotic cells resulting from the cell ruptures and release of the chemotactants into the surrounding tissues [23]. As apoptosis is believed to play a major role in the survival and proliferation of the neoplastic cells, failure to activate apoptosis represents as one of the major obstacles to the success of a particular cancer treatment [24].

### 2.4. Treatment with D/F4 and EA/P2 of *D. Suffruticosa* Induced G_2_/M Phase Cell Cycle Arrest in Cancer Cell Lines

**Figure 4:**
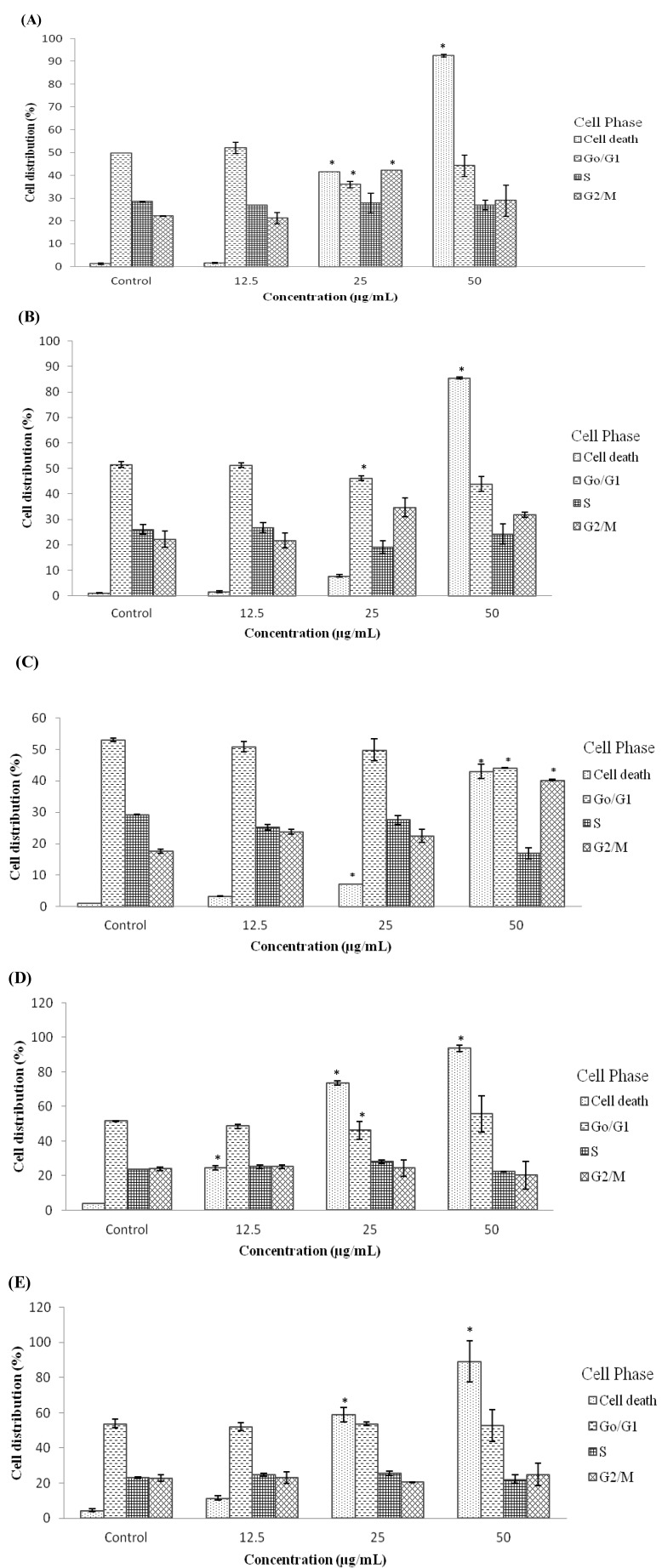
Effects of (**A**) D/F4, (**B**) D/F5 and (**C**) EA/P2 on the cell cycle of MCF-7 and effects of (**D**) D/F4 and (**E**) D/F5 on the cell cycle of MDA-MB-231 analyzed by flow cytometer. The cells were treated with different concentrations of D/F4 for 24 h. * *p* < 0.05 as compared to the untreated control cells. The data is the average value of three independent experiments ± SD.

Different concentrations of the active fractions of DCM (D/F4 and D/F5) and EA (EA/P2) of *D. suffruticosa* extracts were evaluated for the effects on the cell cycle of MCF-7 and MDA-MB-231 cells. As shown in Figure 4A, there was a significant (*p* < 0.05) increase in the population of MCF-7 cells at the G_2_/M phase as compared to the control group after treatment with 25 µg/mL of D/F4 for 24 h. The increment was accompanied by a significant (*p* < 0.05) decrease in the population of MCF-7 cells at the G_o_/G1 phase. The same trend (increase in the population at the G_2_/M phase) was also observed in the MCF-7 cells treated with 50 µg/mL of EA/P2, with significant (*p* < 0.05) decrease in the population at the S and G_o_/G1 phase (Figure 4C). While there was only a significant (*p* < 0.05) increase in the accumulation of MCF-7 cells at the sub-G_1_/cell death phase after treatment with D/F5 to the control group as shown in Figure 4B, without cell cycle arrest. 

Commonly, the cell cycle arrest at G_2_/M is triggered by up-regulation of p53 in response to DNA damage that subsequently inhibits the activation of cdc2-cyclin B complexes. These trigger the dephosphorylation of cyclin dependent kinase (cdk) which in turn leads to cell cycle arrest [3,4]. Nevertheless, the actual mechanisms on how D/F4 and EA/P2 of *D. suffruticosa* caused cell cycle arrest at the G_2_/M checkpoint in MCF-7 cells are still unknown. Previously, many studies have reported the cytotoxic properties of active constituents from plants through G_2_/M arrest [25]. The cell cycle arrest at the G_2_/M phase by chemotherapeutic drugs including doxorubicin [26], irinotecan [27], etoposide [28] and paclitaxel [29] has also been reported.

On the other hand, there was only a significant (*p* < 0.05) increase in the accumulation of MDA-MB-231 cells at the sub-G_1_/cell death phase after treatment with D/F4 and D/F5 as compared to the control group as shown in Figure 4C,D, respectively, without cell cycle arrest. Accumulation of cells at the sub-G_1_ due to cleavage of nuclear DNA into multiple fragments [30] is an indication of induction of cell death through apoptosis [31,32]. Armania *et al.* [18] suggested that the presence of active constituents such as saponins, triterpenes, sterols and polyphenolic compounds in the *D. suffruticosa* extracts contributes to the cytotoxic activity against cancer cell lines.

## 3. Experimental

### 3.1. Plant Material

The whole part of the plant (*D. suffruticosa*) was collected in July, 2009 from the state of Terengganu, Malaysia. Authentication was done at the Biodiversity Unit, Institute of Bioscience, Universiti Putra Malaysia. The voucher specimen was deposited as SK1937/11.

### 3.2. Chemicals

Hexane, dichloromethane (DCM), ethyl acetate (EtOAc), methanol (MeOH), dimethyl sulfoxide (DMSO), ethanol and sulfuric acid were purchased from Fisher Scientific (Loughborough, Leicestershire, UK); 3-[4,5-dimethylthiazol-2-yl]-2,5-diphenyltetrazolium bromide (MTT), RNase A, ferric (III) chloride, phosphate-buffered saline (PBS), trypan blue, propidium iodide (PI) were purchased from Sigma–Aldrich Co. (St. Louis, MO, USA); silica gel 60, 0.063–0.200 mm and thin layer chromatography (TLC) silica gel 60 F254 plates were purchased from Merck (Darmstadt, Germany); RPMI 1640, Mycoplex^TM^ fetal bovine serum (FBS), penicillin and streptomycin (100x), and trypsin EDTA (1×) were purchased from PAA Laboratories GmBH (Pasching, Austria); Annexin V–FITC Apoptosis Detection Kit I was purchased from BD Biosciences Pharmingen (Franklin Lakes, NJ, USA). All the reagents or chemicals used were of analytical grade purity.

### 3.3. Extraction of Plant Material

Previously, DCM and EtOAc extracts of *D. suffruticosa* obtained by sequential solvent extraction has shown better cytotoxic properties as compared to other extracts (hexane and MeOH). Therefore, the extraction procedures were carried out as previously described to obtain DCM and EtOAc extracts [18]. Briefly, the dried root of the plant was powdered using a grinder (Waring^®^, Torrington, CT, USA). Subsequently, 1 kg of the powdered root was macerated with hexane (1:10, w/v) for 72 h at room temperature (25 ± 2 °C) to remove the non-polar fractions. The dried residue was then macerated with DCM (1:10, w/v) and EtOAc (1:10, w/v), sequentially. The procedure for each solvent was repeated twice. The solvent from each extract was removed under reduced pressure (Rotavapor R210, Buchi, Flawil, Switzerland), yielding DCM and EtOAc extracts. The extracts were kept at −20 °C prior to use.

### 3.4. Fractionation of DCM and EtOAc Extract of *D. Suffruticosa*

**Figure 5:**
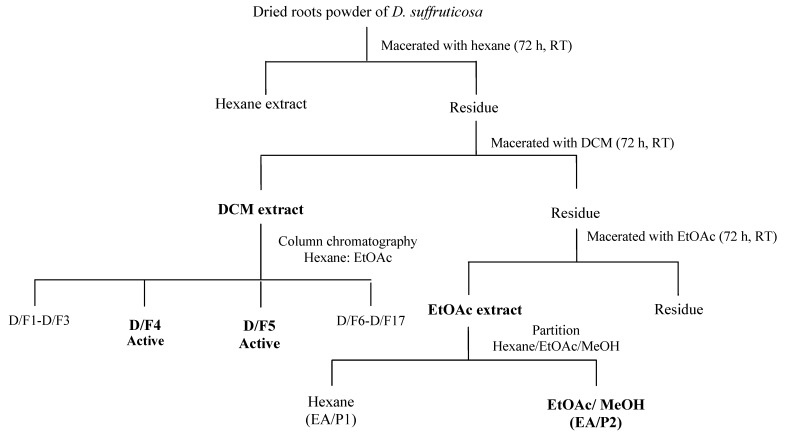
Chromatographic fractionation of DCM and EtOAc extract of *D. suffruticosa* roots. D/F4 and D/F5 represent the active fractions obtained from fractionation of DCM extract using hexane: EtOAc gradient as mobile phase. EA/P2 represents the active fraction obtained from fractionation of EtOAc extract using hexane: EtOAc: MeOH as mobile phase.

The outline for fractionation of DCM and EtOAc extract is shown in Figure 5. Briefly, 2 g of the DCM extract was passed through a column chromatography (3 × 20 cm) packed with silica gel 60, 0.063–0.200 mm and eluted with mobile phase consisted of combination of hexane/EtOAc (100:0–0:100 *v/v*, 500 mL), EtOAc–MeOH (99:1–90:10 *v/v*; 500 mL), and finally changed to MeOH (100% v; 500 mL). The eluent was collected as a fraction of 500 mL for each solvent composition. According to the TLC profile of DCM extract, hexane/EtOAc/MeOH was chosen as the most suitable mobile phase for the fractionation of the extract. The various chemical components present in the fraction were evaluated using TLC with hexane/EtOAc as mobile phase in various proportions (Table 1). The spots were visualized using ultraviolet (UV) light (254 and 365 nm) whereby 10% ethanolic sulfuric acid and 1% ferric (III) chloride were used as staining reagents. The fractions sharing a similar profile were pooled, and a total of 17 fractions were obtained.

Meanwhile, 12.5 g of EtOAc extract was partitioned with hexane (200 mL) and EtOAc/MeOH (1:1 *v/v*, 200 mL) to separate the non-polar and polar compounds present in the extract. The choice of solvent used in the partition process of the extract was according to the TLC profile of the extract. The procedure was repeated for five times until decoloration of the organic solvents (hexane) occurred. The extract was concentrated under reduced pressure by a rotary evaporator (Buchi) to yield 5.3 g of hexane fraction and 6.4 g of EtOAc/MeOH fraction, designated as ethyl acetate partition 1 (EA/P1) and ethyl acetate partition 2 (EA/P2), respectively. All fractions from DCM and EtOAc extracts were subjected to MTT assay for determination of cytotoxic activity as mentioned in Section 3.7. Subsequently, the active fractions were evaluated for induction of apoptosis and their effects on cell cycle.

### 3.5. Cell Culture

Human breast adenocarcinoma (MDA-MB-231, MCF-7), human lung carcinoma (A549), human ovarian adenocarcinoma (CaOV3), human cervical adenocarcinoma (HeLa) and non-tumorigenic cells (3T3 F442A) were cultured in RPMI 1640 medium supplemented with 10% fetal bovine serum (FBS), penicillin (100 µg/mL) and streptomycin (100 µg/mL). The cancer cell lines were purchased from the American Type Culture Collection (ATCC, Manassas, VA, USA), while 3T3 F442A was purchased from the Health Protection Agency (HPA) Culture Collection (Salisbury, England, UK). The cells were maintained and incubated at 37 °C in a humidified atmosphere, 5% CO_2_ atmosphere. The cell viability was determined by staining with 0.25% of trypan blue dye (1:1, *v/v*), and counted by using a haemocytometer viewed under an inverted light microscope (Olympus, Center Valley, PA, USA).

### 3.6. Test Sample Preparation

A stock solution of 30 mg/mL of the tested fraction was prepared by dissolving it with DMSO. Subsequently, working solution of different concentrations was prepared by dissolving the stock solution with culture medium. DMSO only (0.5%, *v/v*) was used as the vehicle control in complete culture medium.

### 3.7. Determination of Cytotoxic Properties

Briefly, the cells (1 × 10^5^ cells/mL) were seeded in 96-well plates (100 µL/well). After incubation for 24 h (cell recovery and attachment), the cells were treated with different concentrations (15–150 µg/mL) of 19 different fractions of *D. suffruticosa* extract (17 fractions and 2 fractions from the DCM and EtOAc extracts, respectively). The incubation was carried out for 72 h. Next, MTT solution (20 µL, 5 mg/mL) was added to each well. After incubation for 4 h at 37 °C in dark, the supernatant was then aspirated out, and DMSO (100 µL) was added to solubilize the insoluble formazan blue crystals. The absorbance was measured at 570 nm using EL×800™ Absorbance Microplate Reader (BioTek Instruments, Inc., Winooski, VT, USA). The concentration which inhibits 50% of the cellular growth (IC_50_) as compared to the control (untreated cells) was calculated by the following equation (1) and interpolation of non-linear regression analysis of concentration of fraction against the percentage of cell growth inhibition:

Cell growth inhibition (%) = [1 − (OD _Treated_/OD _Control (untreated)_)] × 100 (1)

### 3.8. Morphological Changes of Cells Treated with Active Fractions of *D. Suffruticosa*

Briefly, cells of MCF-7 and MDA-MB-231 (3 mL, 1 × 10^5^ cells/mL) were seeded into each well of 6-well plates and incubated for 24 h (cell attachment and recovery). The cells were treated with different concentrations (12.5–50 µg/mL) of active fractions of *D. suffruticosa* [DCM fraction 4 (D/F4), DCM fraction 5 (D/F5) and ethyl acetate partition 2 (EA/P2)] were grown in a 6-well plates for 72 h. The untreated cell (control) was also included. The morphological changes of the cells were observed under an inverted light microscope (Olympus, Center Valley, PA, USA).

### 3.9. Determination of Mode of Cell Death by Annexin V-Propidium Iodide (AnnV-PI)

Mode of cell death induced by the active fractions of *D. suffruticosa* was evaluated by using Annexin V-FITC Apoptosis Detection Kit (BD Biosciences). Briefly, the cells were treated as previously described in Section 3.8. After incubation (24 h), the untreated and treated cells were harvested and washed with cold PBS. Subsequently, the cells were mixed with 190 µL of pre-diluted binding buffer (1×) containing Annexin V-FITC (5 µL) and of PI (10 µL) (1 mg/mL) and further incubated for 10 min at 37 °C in the dark. Subsequently, 300 µL of binding buffer (1×) was added into each tube. The percentage of cell undergoing apoptosis and necrosis was quantified using a flow cytometer (Becton Dickinson, Franklin Lakes, NJ, USA) equipped with Cell Quest software within 1 h.

### 3.10. Determination of Cell Cycle Arrest by Flow Cytometer

The cells were treated as previously described in Section 3.8. Untreated cells (control) were also included. After incubation (24 h), the cells were harvested and fixed with ice-cold 70% ethanol (1 mL) at −20 °C for 2 h. Prior to analysis, the cells were washed with cold PBS and re-suspended in 425 µL of PBS, 25 µL PI (1 mg/mL) and 50 µL RNase A (1 mg/mL). The DNA contents were recorded by a flow cytometer (Becton Dickinson) equipped with Cell Quest software.

### 3.11. Statistical Analysis

Data are presented as mean ± S.D (standard deviation) of three independent experiments. The analyses of variance were conducted by using SPSS (Statistical Program for Social Sciences version 20 SPSS Inc, Chicago, DE USA) to evaluate the significant difference. Subsequently, the significance of the difference was evaluated using Dunnet test, and *p* < 0.05 was considered significant.

## 4. Conclusion

In conclusion, it is suggested that the cytotoxicity of the active fractions from *D. suffruticosa* extract in MCF-7 cells was due to G_2_/M cell cycle arrest and induction of apoptosis. While the experimental results are exciting, there are still a few limitations especially on the active compounds of the active fractions. Therefore, further work on isolation and elucidation of bioactive compound(s) that contribute to the cytotoxic properties of *D. suffruticosa* is vital. Besides that, further studies on the mechanisms underlying the induction of apoptosis and cell cycle arrest by the plant extract need to be carried out.
